# 1-(1-Carboxy­methyl-1,4-anhydro-2,3-*O*-isopropyl­idene-α-d-erythrofuranos­yl)thymine

**DOI:** 10.1107/S1600536810001704

**Published:** 2010-01-20

**Authors:** G. M. J. Lenagh-Snow, S. F. Jenkinson, A. J. Stewart, G. W. J. Fleet, D. J. Watkin

**Affiliations:** a Department of Organic Chemistry, Chemistry Research Laboratory, University of Oxford, Mansfield Road, Oxford OX1 3TA, England; b Idenix Pharmaceuticals, Inc., 60 Hampshire Street, Cambridge, MA 02139, USA; c Department of Chemical Crystallography, Chemistry Research Laboratory, University of Oxford, Mansfield Road, Oxford OX1 3TA, England

## Abstract

X-Ray crystallography unequivocally determined the stereochemistry of the thymine base in the title compound, C_14_H_18_N_2_O_7_. The absolute stereochemistry was determined from the use of d-ribose as the starting material. There are two independent mol­ecules in the asymmetric unit (*Z*′ = 2) which exist as N—H⋯O hydrogen-bonded pairs in the crystal structure.

## Related literature

The title compound was obtained during studies on the synthesis of the 5-carbon analogue of psicofuran­ine, a naturally occurring nucleoside. For related literature on psicofuran­ine, see: Schroeder & Hoeksema (1959 ▶); Smith *et al.* (1973 ▶); Garrett (1960 ▶). For anomeric bromination see: Probert *et al.* (2005 ▶); Smith *et al.* (1999 ▶). For the extiction correction, see: Larson (1970 ▶).
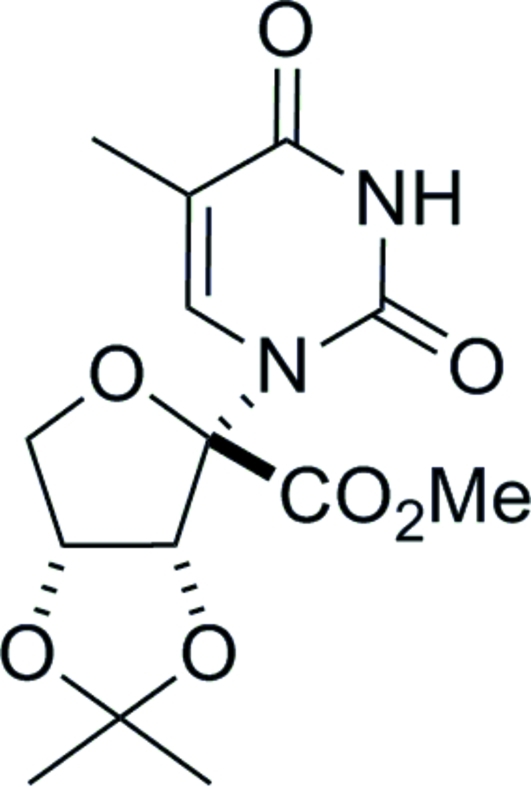


## Experimental

### 

#### Crystal data

C_14_H_18_N_2_O_7_
*M* *_r_* = 326.31
Monoclinic, 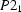

*a* = 7.8937 (5) Å
*b* = 13.3471 (10) Å
*c* = 14.9208 (10) Å
β = 103.565 (4)°
*V* = 1528.17 (18) Å^3^
*Z* = 4
Mo *K*α radiation
μ = 0.12 mm^−1^
*T* = 150 K
0.40 × 0.20 × 0.03 mm

#### Data collection

Nonius KappaCCD diffractometer
Absorption correction: multi-scan (*DENZO*/*SCALEPACK*; Otwinowski & Minor, 1997 ▶) *T*_min_ = 0.83, *T*_max_ = 1.00
9120 measured reflections
3090 independent reflections
2453 reflections with *I* > 2σ(*I*)
*R*_int_ = 0.065

#### Refinement

*R*[*F* ^2^ > 2σ(*F* ^2^)] = 0.047
*wR*(*F* ^2^) = 0.114
*S* = 0.95
3090 reflections
416 parameters
1 restraint
H-atom parameters constrained
Δρ_max_ = 0.34 e Å^−3^
Δρ_min_ = −0.31 e Å^−3^

### 

Data collection: *COLLECT* (Nonius, 2001 ▶); cell refinement: *DENZO*/*SCALEPACK* (Otwinowski & Minor, 1997 ▶); data reduction: *DENZO*/*SCALEPACK*; program(s) used to solve structure: *SIR92* (Altomare *et al.*, 1994 ▶); program(s) used to refine structure: *CRYSTALS* (Betteridge *et al.*, 2003 ▶); molecular graphics: *CAMERON* (Watkin *et al.*, 1996 ▶); software used to prepare material for publication: *CRYSTALS*.

## Supplementary Material

Crystal structure: contains datablocks global, I. DOI: 10.1107/S1600536810001704/lh2979sup1.cif

Structure factors: contains datablocks I. DOI: 10.1107/S1600536810001704/lh2979Isup2.hkl

Additional supplementary materials:  crystallographic information; 3D view; checkCIF report

## Figures and Tables

**Table 1:** Hydrogen-bond geometry (Å, °)

*D*—H⋯*A*	*D*—H	H⋯*A*	*D*⋯*A*	*D*—H⋯*A*
N26—H261⋯O1	0.88	1.93	2.791 (6)	164
N3—H31⋯O24	0.88	2.01	2.863 (6)	165

**Fig. 1.**
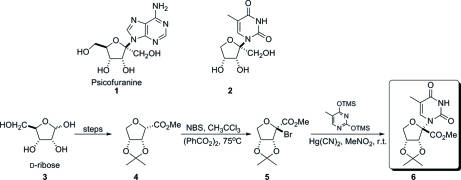
Synthetic Scheme

**Fig. 2.**
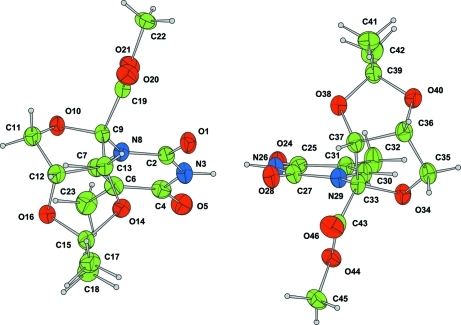
The asymmetric unit of the title compound with displacement ellipsoids drawn at the 50% probability level. H atoms are shown as spheres of arbitary radius.

**Fig. 3.**
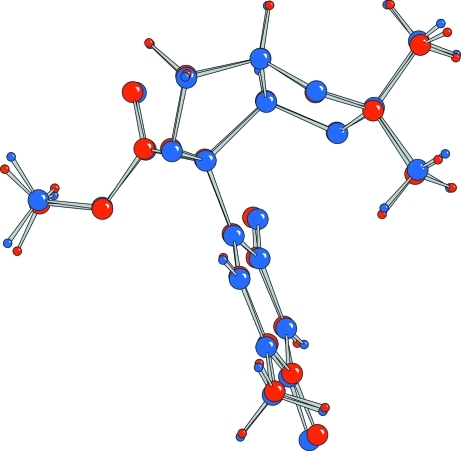
Overlay of the two molecules in the asymmetric unit.

**Fig. 4.**
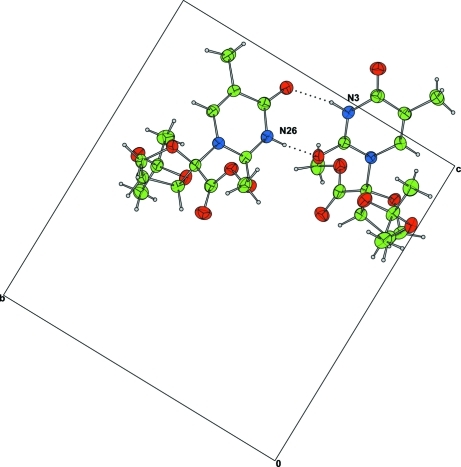
Hydrogen bonded dimer repeating unit. Hydrogen bonds are shown by dotted lines.

**Fig. 5.**
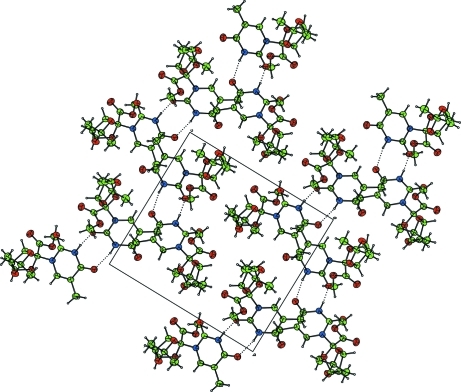
Packing diagram projected along the a-axis. Hydrogen bonds are shown by dotted lines.

## supplementary crystallographic information

### Comment

Nucleosides are a powerful class of anti-viral and anti-bacterial agents. Psicofuranine 1 (Fig. 1) is a naturally occurring nucleoside with a branch at the anomeric position of the sugar (Schroeder & Hoeksema, 1959). It has potent anti-bacterial and anti-tumour activity but is cardiotoxic in man (Smith *et al.*, 1973). Psicofuranine is also unstable in acidic and basic conditions with the *N*-glycosidic bond readily undergoing hydrolytic cleavage (Garrett, 1960). During studies on the synthesis of the 5-carbon analogue of psicofuranine 2 the ester 4 was synthesized. Anomeric radical bromination (Smith *et al.*, 1999) gave rise to a single isolable bromide 5 (Probert *et al.*, 2005) which on displacement with silylated thymine gave a single nucleoside product. The stereochemistry at the anomeric position of the sugar was firmly established by X-ray crystallography and the structure was confirmed as 6 in which the thymine is in the α rather than the desired β position.

There are two crystallographically distinct molecules in the asymmetric unit which are related by a pseudo 2-fold rotation axis (Fig 2). When the two molecules are mapped they show good overlap (Fig. 3) with RMS deviations of 0.1055 on the positions, 0.082 for the bonds and 3.8892 for the torsion angles. These two molecules form hydrogen bonded pairs in the crystal structure (Fig. 4, Fig. 5). In both cases the central nitrogen (N3, N26) between the two carbonyls of the thymine acts as the donor but hydrogen bonds are formed to different carbonyls of the two thymine rings. The absolute stereochemistry was determined from the use of D-ribose as the starting material. Only classical hydrogen bonding was considered.

### Experimental

The title compound was recrystallized by diffusion from a mixture of methanol and acetone: m.p. 457–458 K; [α]_D_^25^ -235.2 (*c*, 0.84 in CHCl_3_).

### Refinement

In the absence of significant anomalous scattering, Friedel pairs were merged and the absolute configuration was assigned from the use of D-ribose as the starting material.

The H atoms were all located in a difference map, but those attached to carbon atoms were repositioned geometrically. The H atoms were initially refined with soft restraints on the bond lengths and angles to regularize their geometry (C—H in the range 0.93–0.98, N—H in the range 0.86–0.89 N—H to 0.86 O—H = 0.82 Å) and *U*_iso_(H) (in the range 1.2–1.5 times *U*_eq_ of the parent atom), after which the positions were refined with riding constraints.

### Crystal data

**Table:**

C_14_H_18_N_2_O_7_	*F*(000) = 688
*M**_r_* = 326.31	*D*_x_ = 1.418 Mg m^−^^3^
Monoclinic, *P*2_1_	Mo *K*α radiation, λ = 0.71073 Å
Hall symbol: P 2yb	Cell parameters from 2763 reflections
*a* = 7.8937 (5) Å	θ = 5–26°
*b* = 13.3471 (10) Å	µ = 0.12 mm^−^^1^
*c* = 14.9208 (10) Å	*T* = 150 K
β = 103.565 (4)°	Plate, colourless
*V* = 1528.17 (18) Å^3^	0.40 × 0.20 × 0.03 mm
*Z* = 4	

### Data collection

**Table:**

Nonius KappaCCD diffractometer	2453 reflections with *I* > 2σ(*I*)
graphite	*R*_int_ = 0.065
ω scans	θ_max_ = 26.1°, θ_min_ = 5.2°
Absorption correction: multi-scan (*DENZO*/*SCALEPACK*; Otwinowski & Minor, 1997)	*h* = −9→9
*T*_min_ = 0.83, *T*_max_ = 1.00	*k* = −14→16
9120 measured reflections	*l* = −18→18
3090 independent reflections	

### Refinement

**Table:**

Refinement on *F*^2^	Hydrogen site location: inferred from neighbouring sites
Least-squares matrix: full	H-atom parameters constrained
*R*[*F*^2^ > 2σ(*F*^2^)] = 0.047	Method = Modified Sheldrick *w* = 1/[σ^2^(*F*^2^) + (0.04*P*)^2^ + 0.5*P*], where *P* = (max(*F*_o_^2^,0) + 2*F*_c_^2^)/3
*wR*(*F*^2^) = 0.114	(Δ/σ)_max_ = 0.0002
*S* = 0.95	Δρ_max_ = 0.34 e Å^−^^3^
3090 reflections	Δρ_min_ = −0.31 e Å^−^^3^
416 parameters	Extinction correction: Larson (1970), Equation 22
1 restraint	Extinction coefficient: 590 (70)
Primary atom site location: structure-invariant direct methods	

### Fractional atomic coordinates and isotropic or equivalent isotropic displacement parameters (Å^2^)

**Table:**

	*x*	*y*	*z*	*U*_iso_*/*U*_eq_	
O1	0.2931 (4)	0.3816 (2)	0.81712 (19)	0.0363	
C2	0.3274 (5)	0.3347 (3)	0.8903 (3)	0.0308	
N3	0.3044 (5)	0.3728 (3)	0.9715 (2)	0.0346	
C4	0.3343 (6)	0.3220 (3)	1.0559 (3)	0.0348	
O5	0.3097 (4)	0.3655 (2)	1.1245 (2)	0.0414	
C6	0.3964 (5)	0.2203 (3)	1.0536 (3)	0.0336	
C7	0.4220 (5)	0.1836 (3)	0.9739 (3)	0.0321	
N8	0.3912 (4)	0.2388 (2)	0.8940 (2)	0.0290	
C9	0.4110 (5)	0.1982 (3)	0.8058 (3)	0.0292	
O10	0.5057 (4)	0.10739 (19)	0.82495 (18)	0.0320	
C11	0.4357 (5)	0.0365 (3)	0.7525 (3)	0.0348	
C12	0.2451 (5)	0.0597 (3)	0.7235 (3)	0.0343	
C13	0.2338 (5)	0.1728 (3)	0.7383 (3)	0.0320	
O14	0.0903 (4)	0.1860 (2)	0.7798 (2)	0.0374	
C15	0.0163 (5)	0.0881 (3)	0.7891 (3)	0.0356	
O16	0.1544 (4)	0.0203 (2)	0.7876 (2)	0.0362	
C17	−0.1363 (6)	0.0703 (3)	0.7081 (3)	0.0455	
C18	−0.0325 (6)	0.0826 (4)	0.8799 (3)	0.0469	
C19	0.5238 (5)	0.2679 (3)	0.7618 (3)	0.0324	
O20	0.5064 (4)	0.2774 (2)	0.6798 (2)	0.0417	
O21	0.6543 (4)	0.3076 (2)	0.8269 (2)	0.0380	
C22	0.7785 (6)	0.3686 (4)	0.7933 (3)	0.0443	
C23	0.4312 (7)	0.1600 (4)	1.1418 (3)	0.0465	
O24	0.2166 (4)	0.5799 (2)	0.93916 (19)	0.0374	
C25	0.1273 (5)	0.6136 (3)	0.8657 (3)	0.0313	
N26	0.0825 (5)	0.5521 (2)	0.7891 (2)	0.0323	
C27	−0.0261 (5)	0.5758 (3)	0.7053 (3)	0.0310	
O28	−0.0626 (4)	0.5168 (2)	0.64081 (19)	0.0375	
N29	−0.0943 (5)	0.6715 (2)	0.6997 (2)	0.0306	
C30	−0.0454 (5)	0.7396 (3)	0.7711 (3)	0.0326	
C31	0.0618 (5)	0.7156 (3)	0.8524 (3)	0.0305	
C32	0.1154 (7)	0.7889 (3)	0.9308 (3)	0.0461	
C33	−0.2093 (5)	0.6978 (3)	0.6101 (3)	0.0305	
O34	−0.2864 (4)	0.7906 (2)	0.62183 (18)	0.0339	
C35	−0.3007 (6)	0.8493 (3)	0.5385 (3)	0.0351	
C36	−0.1471 (5)	0.8204 (3)	0.5013 (3)	0.0333	
C37	−0.1111 (5)	0.7103 (3)	0.5318 (3)	0.0339	
O38	0.0732 (4)	0.7037 (2)	0.5655 (2)	0.0364	
C39	0.1460 (6)	0.7998 (3)	0.5517 (3)	0.0354	
O40	0.0075 (4)	0.8694 (2)	0.5505 (2)	0.0368	
C41	0.2020 (6)	0.8002 (4)	0.4618 (3)	0.0415	
C42	0.2914 (6)	0.8224 (4)	0.6342 (3)	0.0473	
C43	−0.3599 (6)	0.6221 (3)	0.5830 (3)	0.0347	
O44	−0.4208 (4)	0.5954 (2)	0.65563 (19)	0.0351	
C45	−0.5767 (6)	0.5327 (3)	0.6364 (3)	0.0430	
O46	−0.4246 (4)	0.5982 (2)	0.5041 (2)	0.0448	
H71	0.4624	0.1181	0.9722	0.0371*	
H111	0.4513	−0.0316	0.7764	0.0408*	
H112	0.4950	0.0448	0.7026	0.0412*	
H121	0.1912	0.0372	0.6595	0.0412*	
H131	0.2163	0.2111	0.6801	0.0380*	
H172	−0.1903	0.0060	0.7168	0.0643*	
H173	−0.0963	0.0702	0.6514	0.0642*	
H171	−0.2232	0.1235	0.7066	0.0639*	
H181	−0.0780	0.0161	0.8871	0.0748*	
H183	0.0704	0.0951	0.9291	0.0752*	
H182	−0.1213	0.1339	0.8809	0.0750*	
H222	0.8569	0.3999	0.8455	0.0703*	
H221	0.8444	0.3287	0.7598	0.0705*	
H223	0.7183	0.4210	0.7528	0.0699*	
H232	0.4644	0.0921	1.1292	0.0681*	
H233	0.3276	0.1583	1.1672	0.0683*	
H231	0.5292	0.1900	1.1863	0.0683*	
H301	−0.0922	0.8051	0.7627	0.0356*	
H323	0.0689	0.8550	0.9114	0.0654*	
H322	0.2425	0.7918	0.9490	0.0654*	
H321	0.0697	0.7671	0.9830	0.0652*	
H351	−0.4091	0.8324	0.4936	0.0382*	
H352	−0.2992	0.9212	0.5538	0.0381*	
H361	−0.1674	0.8283	0.4335	0.0381*	
H371	−0.1535	0.6632	0.4807	0.0422*	
H412	0.2448	0.8668	0.4522	0.0641*	
H411	0.2934	0.7499	0.4646	0.0643*	
H413	0.1026	0.7836	0.4127	0.0639*	
H421	0.3481	0.8846	0.6256	0.0668*	
H422	0.3738	0.7673	0.6428	0.0670*	
H423	0.2421	0.8276	0.6879	0.0672*	
H453	−0.6176	0.5238	0.6929	0.0613*	
H452	−0.5496	0.4674	0.6141	0.0609*	
H451	−0.6673	0.5654	0.5897	0.0615*	
H261	0.1302	0.4919	0.7947	0.0383*	
H31	0.2694	0.4353	0.9707	0.0413*	

### Atomic displacement parameters (Å^2^)

**Table:**

	*U*^11^	*U*^22^	*U*^33^	*U*^12^	*U*^13^	*U*^23^
O1	0.0473 (17)	0.0277 (14)	0.0351 (16)	0.0081 (13)	0.0120 (14)	0.0074 (13)
C2	0.034 (2)	0.0255 (19)	0.033 (2)	0.0003 (17)	0.0092 (18)	0.0010 (18)
N3	0.0444 (19)	0.0259 (15)	0.0338 (19)	0.0032 (16)	0.0095 (16)	−0.0002 (15)
C4	0.034 (2)	0.036 (2)	0.035 (2)	−0.0036 (19)	0.0083 (19)	−0.0014 (19)
O5	0.0476 (18)	0.0436 (17)	0.0347 (16)	−0.0022 (16)	0.0128 (14)	−0.0064 (15)
C6	0.036 (2)	0.035 (2)	0.030 (2)	−0.0005 (19)	0.0074 (18)	0.0005 (18)
C7	0.031 (2)	0.032 (2)	0.032 (2)	0.0015 (17)	0.0062 (18)	0.0057 (17)
N8	0.0345 (17)	0.0266 (16)	0.0267 (17)	0.0057 (14)	0.0090 (14)	0.0010 (13)
C9	0.034 (2)	0.0221 (18)	0.031 (2)	0.0012 (17)	0.0079 (18)	0.0014 (16)
O10	0.0344 (14)	0.0229 (13)	0.0370 (15)	0.0019 (12)	0.0052 (12)	−0.0029 (12)
C11	0.036 (2)	0.029 (2)	0.040 (2)	−0.0002 (19)	0.0102 (19)	−0.0074 (19)
C12	0.035 (2)	0.031 (2)	0.037 (2)	0.0000 (18)	0.0094 (19)	−0.0027 (18)
C13	0.037 (2)	0.028 (2)	0.031 (2)	0.0029 (18)	0.0078 (18)	0.0004 (17)
O14	0.0329 (16)	0.0322 (15)	0.0482 (18)	0.0012 (13)	0.0122 (14)	−0.0035 (13)
C15	0.032 (2)	0.0252 (19)	0.048 (3)	−0.0016 (18)	0.0082 (19)	−0.0045 (19)
O16	0.0344 (16)	0.0272 (14)	0.0482 (18)	0.0020 (13)	0.0122 (14)	0.0032 (13)
C17	0.033 (2)	0.043 (3)	0.059 (3)	−0.002 (2)	0.008 (2)	−0.007 (2)
C18	0.044 (3)	0.043 (2)	0.057 (3)	0.001 (2)	0.018 (2)	0.001 (2)
C19	0.032 (2)	0.0254 (19)	0.041 (2)	0.0035 (18)	0.010 (2)	0.0018 (18)
O20	0.0489 (19)	0.0462 (18)	0.0318 (16)	−0.0022 (15)	0.0131 (15)	0.0039 (14)
O21	0.0370 (16)	0.0353 (15)	0.0405 (16)	−0.0056 (14)	0.0070 (14)	0.0041 (13)
C22	0.034 (2)	0.041 (2)	0.059 (3)	−0.009 (2)	0.013 (2)	0.010 (2)
C23	0.055 (3)	0.049 (3)	0.036 (2)	0.009 (2)	0.011 (2)	0.008 (2)
O24	0.0423 (17)	0.0327 (15)	0.0333 (15)	0.0038 (14)	0.0012 (14)	0.0006 (13)
C25	0.029 (2)	0.031 (2)	0.033 (2)	−0.0001 (18)	0.0057 (18)	0.0009 (18)
N26	0.0386 (19)	0.0253 (16)	0.0315 (19)	0.0029 (15)	0.0055 (16)	−0.0003 (14)
C27	0.036 (2)	0.0234 (18)	0.034 (2)	−0.0007 (17)	0.0077 (18)	−0.0008 (17)
O28	0.0499 (18)	0.0265 (14)	0.0350 (16)	0.0015 (14)	0.0078 (14)	−0.0022 (13)
N29	0.0365 (19)	0.0266 (16)	0.0277 (17)	0.0020 (15)	0.0054 (15)	−0.0005 (14)
C30	0.037 (2)	0.0256 (19)	0.034 (2)	0.0014 (17)	0.0056 (19)	−0.0034 (17)
C31	0.035 (2)	0.0270 (19)	0.029 (2)	0.0006 (18)	0.0081 (18)	−0.0037 (17)
C32	0.056 (3)	0.033 (2)	0.043 (3)	0.004 (2)	−0.002 (2)	−0.007 (2)
C33	0.038 (2)	0.0236 (18)	0.030 (2)	0.0053 (17)	0.0075 (18)	0.0003 (16)
O34	0.0431 (17)	0.0279 (14)	0.0313 (14)	0.0063 (13)	0.0101 (14)	0.0035 (12)
C35	0.041 (2)	0.029 (2)	0.033 (2)	0.0036 (19)	0.0027 (19)	0.0061 (17)
C36	0.035 (2)	0.030 (2)	0.031 (2)	0.0022 (18)	0.0020 (18)	0.0027 (17)
C37	0.040 (2)	0.0294 (19)	0.032 (2)	−0.0002 (19)	0.0082 (19)	−0.0040 (18)
O38	0.0371 (16)	0.0274 (14)	0.0451 (17)	0.0044 (13)	0.0107 (14)	0.0032 (13)
C39	0.043 (3)	0.0258 (19)	0.039 (2)	0.0027 (19)	0.013 (2)	0.0005 (18)
O40	0.0356 (15)	0.0291 (13)	0.0456 (17)	0.0016 (13)	0.0092 (14)	−0.0033 (13)
C41	0.041 (3)	0.046 (2)	0.040 (2)	−0.002 (2)	0.016 (2)	0.002 (2)
C42	0.041 (3)	0.050 (3)	0.048 (3)	0.004 (2)	0.007 (2)	−0.009 (2)
C43	0.038 (2)	0.030 (2)	0.036 (2)	0.0048 (19)	0.008 (2)	−0.0004 (18)
O44	0.0372 (16)	0.0325 (15)	0.0353 (15)	−0.0068 (13)	0.0081 (13)	−0.0031 (13)
C45	0.045 (3)	0.033 (2)	0.051 (3)	−0.011 (2)	0.012 (2)	−0.001 (2)
O46	0.0479 (18)	0.0505 (18)	0.0323 (15)	−0.0102 (16)	0.0022 (14)	−0.0051 (16)

### Geometric parameters (Å, °)

**Table:**

O1—C2	1.233 (5)	O24—C25	1.240 (5)
C2—N3	1.365 (5)	C25—N26	1.384 (5)
C2—N8	1.371 (5)	C25—C31	1.453 (5)
N3—C4	1.401 (5)	N26—C27	1.377 (5)
N3—H31	0.877	N26—H261	0.882
C4—O5	1.230 (5)	C27—O28	1.224 (5)
C4—C6	1.448 (6)	C27—N29	1.380 (5)
C6—C7	1.345 (6)	N29—C30	1.385 (5)
C6—C23	1.512 (6)	N29—C33	1.471 (5)
C7—N8	1.374 (5)	C30—C31	1.346 (5)
C7—H71	0.933	C30—H301	0.946
N8—C9	1.464 (5)	C31—C32	1.507 (6)
C9—O10	1.418 (5)	C32—H323	0.973
C9—C13	1.557 (6)	C32—H322	0.977
C9—C19	1.537 (5)	C32—H321	0.977
O10—C11	1.445 (5)	C33—O34	1.408 (5)
C11—C12	1.497 (6)	C33—C37	1.555 (5)
C11—H111	0.974	C33—C43	1.541 (6)
C11—H112	0.974	O34—C35	1.452 (5)
C12—C13	1.532 (5)	C35—C36	1.497 (6)
C12—O16	1.423 (5)	C35—H351	0.981
C12—H121	0.996	C35—H352	0.986
C13—O14	1.424 (5)	C36—C37	1.544 (6)
C13—H131	0.988	C36—O40	1.427 (5)
O14—C15	1.452 (5)	C36—H361	0.992
C15—O16	1.420 (5)	C37—O38	1.426 (5)
C15—C17	1.512 (6)	C37—H371	0.985
C15—C18	1.496 (6)	O38—C39	1.440 (5)
C17—H172	0.981	C39—O40	1.431 (5)
C17—H173	0.969	C39—C41	1.507 (6)
C17—H171	0.984	C39—C42	1.504 (6)
C18—H181	0.974	C41—H412	0.974
C18—H183	0.973	C41—H411	0.979
C18—H182	0.982	C41—H413	0.965
C19—O20	1.205 (5)	C42—H421	0.966
C19—O21	1.348 (5)	C42—H422	0.971
O21—C22	1.451 (5)	C42—H423	0.972
C22—H222	0.969	C43—O44	1.333 (5)
C22—H221	0.962	C43—O46	1.210 (5)
C22—H223	0.972	O44—C45	1.460 (5)
C23—H232	0.974	C45—H453	0.979
C23—H233	0.979	C45—H452	0.975
C23—H231	0.979	C45—H451	0.977
			
O1—C2—N3	123.3 (4)	O24—C25—N26	119.7 (4)
O1—C2—N8	120.7 (3)	O24—C25—C31	124.8 (4)
N3—C2—N8	115.9 (3)	N26—C25—C31	115.5 (3)
C2—N3—C4	126.0 (3)	C25—N26—C27	126.7 (3)
C2—N3—H31	116.7	C25—N26—H261	116.2
C4—N3—H31	117.3	C27—N26—H261	117.2
N3—C4—O5	119.6 (4)	N26—C27—O28	123.2 (3)
N3—C4—C6	114.8 (4)	N26—C27—N29	114.8 (3)
O5—C4—C6	125.6 (4)	O28—C27—N29	122.0 (4)
C4—C6—C7	119.0 (4)	C27—N29—C30	121.7 (3)
C4—C6—C23	118.2 (4)	C27—N29—C33	115.2 (3)
C7—C6—C23	122.8 (4)	C30—N29—C33	122.9 (3)
C6—C7—N8	122.6 (4)	N29—C30—C31	122.7 (4)
C6—C7—H71	119.2	N29—C30—H301	118.4
N8—C7—H71	118.2	C31—C30—H301	118.9
C7—N8—C2	121.6 (3)	C25—C31—C30	118.3 (4)
C7—N8—C9	123.1 (3)	C25—C31—C32	118.5 (4)
C2—N8—C9	115.2 (3)	C30—C31—C32	123.2 (4)
N8—C9—O10	107.4 (3)	C31—C32—H323	110.0
N8—C9—C13	113.1 (3)	C31—C32—H322	108.9
O10—C9—C13	107.2 (3)	H323—C32—H322	109.8
N8—C9—C19	110.8 (3)	C31—C32—H321	109.6
O10—C9—C19	105.8 (3)	H323—C32—H321	108.8
C13—C9—C19	112.2 (3)	H322—C32—H321	109.7
C9—O10—C11	108.5 (3)	N29—C33—O34	106.9 (3)
O10—C11—C12	105.3 (3)	N29—C33—C37	113.4 (3)
O10—C11—H111	110.1	O34—C33—C37	107.8 (3)
C12—C11—H111	109.3	N29—C33—C43	110.8 (3)
O10—C11—H112	109.2	O34—C33—C43	106.3 (3)
C12—C11—H112	112.7	C37—C33—C43	111.2 (3)
H111—C11—H112	110.1	C33—O34—C35	108.5 (3)
C11—C12—C13	104.5 (3)	O34—C35—C36	105.7 (3)
C11—C12—O16	111.1 (3)	O34—C35—H351	109.9
C13—C12—O16	102.2 (3)	C36—C35—H351	109.9
C11—C12—H121	112.9	O34—C35—H352	109.4
C13—C12—H121	114.1	C36—C35—H352	111.7
O16—C12—H121	111.4	H351—C35—H352	110.1
C12—C13—C9	103.5 (3)	C35—C36—C37	104.2 (3)
C12—C13—O14	105.3 (3)	C35—C36—O40	111.1 (3)
C9—C13—O14	112.2 (3)	C37—C36—O40	102.1 (3)
C12—C13—H131	112.6	C35—C36—H361	113.5
C9—C13—H131	112.0	C37—C36—H361	112.1
O14—C13—H131	110.9	O40—C36—H361	112.9
C13—O14—C15	108.0 (3)	C36—C37—C33	103.5 (3)
O14—C15—O16	104.2 (3)	C36—C37—O38	105.3 (3)
O14—C15—C17	109.1 (3)	C33—C37—O38	112.1 (3)
O16—C15—C17	111.0 (3)	C36—C37—H371	111.9
O14—C15—C18	109.0 (3)	C33—C37—H371	112.0
O16—C15—C18	110.2 (4)	O38—C37—H371	111.5
C17—C15—C18	113.0 (4)	C37—O38—C39	107.7 (3)
C12—O16—C15	106.6 (3)	O38—C39—O40	104.5 (3)
C15—C17—H172	108.7	O38—C39—C41	109.9 (3)
C15—C17—H173	109.6	O40—C39—C41	111.7 (3)
H172—C17—H173	110.8	O38—C39—C42	108.4 (4)
C15—C17—H171	109.0	O40—C39—C42	108.3 (3)
H172—C17—H171	108.2	C41—C39—C42	113.7 (4)
H173—C17—H171	110.5	C39—O40—C36	105.5 (3)
C15—C18—H181	108.9	C39—C41—H412	108.6
C15—C18—H183	109.1	C39—C41—H411	109.1
H181—C18—H183	109.7	H412—C41—H411	110.7
C15—C18—H182	108.4	C39—C41—H413	108.6
H181—C18—H182	110.6	H412—C41—H413	110.2
H183—C18—H182	110.0	H411—C41—H413	109.6
C9—C19—O20	123.9 (4)	C39—C42—H421	110.6
C9—C19—O21	110.5 (3)	C39—C42—H422	108.4
O20—C19—O21	125.2 (4)	H421—C42—H422	110.6
C19—O21—C22	115.8 (3)	C39—C42—H423	108.2
O21—C22—H222	108.7	H421—C42—H423	109.6
O21—C22—H221	111.1	H422—C42—H423	109.4
H222—C22—H221	109.6	C33—C43—O44	111.4 (3)
O21—C22—H223	110.3	C33—C43—O46	123.5 (4)
H222—C22—H223	108.4	O44—C43—O46	124.8 (4)
H221—C22—H223	108.7	C43—O44—C45	116.2 (3)
C6—C23—H232	109.2	O44—C45—H453	109.5
C6—C23—H233	110.6	O44—C45—H452	109.7
H232—C23—H233	109.9	H453—C45—H452	109.2
C6—C23—H231	109.0	O44—C45—H451	109.1
H232—C23—H231	107.9	H453—C45—H451	109.6
H233—C23—H231	110.3	H452—C45—H451	109.8

### Hydrogen-bond geometry (Å, °)

**Table:**

*D*—H···*A*	*D*—H	H···*A*	*D*···*A*	*D*—H···*A*
C11—H111···O5^i^	0.97	2.52	3.301 (6)	138
N26—H261···O1	0.88	1.93	2.791 (6)	164
N3—H31···O24	0.88	2.01	2.863 (6)	165

Symmetry codes: (i) −*x*+1, *y*−1/2, −*z*+2.

## References

[bb1] Altomare, A., Cascarano, G., Giacovazzo, C., Guagliardi, A., Burla, M. C., Polidori, G. & Camalli, M. (1994). *J. Appl. Cryst.* **27**, 435.

[bb2] Betteridge, P. W., Carruthers, J. R., Cooper, R. I., Prout, K. & Watkin, D. J. (2003). *J. Appl. Cryst.* **36**, 1487.

[bb3] Garrett, E. R. (1960). *J. Am. Chem. Soc.* **82**, 827–832.

[bb4] Larson, A. C. (1970). *Crystallographic Computing*, edited by F. R. Ahmed, S. R. Hall & C. P. Huber, pp. 291–294. Copenhagen: Munksgaard.

[bb5] Nonius (2001). *COLLECT* Nonius BV, Delft, The Netherlands.

[bb6] Otwinowski, Z. & Minor, W. (1997). *Methods in Enzymology*, Vol. 276, *Macromolecular Crystallography*, Part A, edited by C. W. Carter Jr & R. M. Sweet, pp. 307–326. New York: Academic Press.

[bb7] Probert, M. R., Watkin, D. J., Stewart, A. J., Storer, R. & Fleet, G. W. J. (2005). *Acta Cryst.* E**61**, o1718–o1720.

[bb8] Schroeder, W. & Hoeksema, H. (1959). *J. Am. Chem. Soc.* **81**, 1767–1768.

[bb9] Smith, M. D., Long, D. D., Martín, A., Campbell, N., Blériot, Y. & Fleet, G. W. J. (1999). *Synlett*, **7**, 1151–1154.

[bb10] Smith, C. G., Poutsiaka, J. W. & Schreiber, E. C. (1973). *J. Int. Med. Res.* **1**, 489–503.

[bb11] Watkin, D. J., Prout, C. K. & Pearce, L. J. (1996). *CAMERON* Chemical Crystallography Laboratory, Oxford, England.

